# Self-Reported Parental Healthy Dietary Behavior Relates to Views on Child Feeding and Health and Diet Quality

**DOI:** 10.3390/nu15041024

**Published:** 2023-02-18

**Authors:** Irene Mäkelä, Ella Koivuniemi, Tero Vahlberg, Monique M. Raats, Kirsi Laitinen

**Affiliations:** 1 Institute of Biomedicine, Research Centre for Integrative Physiology and Pharmacology, Faculty of Medicine, University of Turku, 20014 Turku, Finland; 2 Institute of Clinical Medicine, Biostatistics, Faculty of Medicine, University of Turku, 20014 Turku, Finland; 3 Food, Consumer Behaviour and Health (FCBH) Research Centre, School of Psychology, Faculty of Health and Medical Sciences, University of Surrey, Guildford GU2 7XH, UK; 4 Functional Foods Forum, Faculty of Medicine, University of Turku, 20014 Turku, Finland; 5 Department of Obstetrics and Gynecology, Turku University Hospital, 20521 Turku, Finland

**Keywords:** preschool children, health-consciousness, parents, child feeding

## Abstract

The aim of this cross-sectional study was to investigate whether parental views on child feeding and its impact on health differ between those parents whose self-perception was that they followed a healthy diet to those who do not. Furthermore, differences in the child’s diet quality and weight were compared between the groups. Parents of 2−6-year-old children (*n* = 738), recruited from child health clinics throughout Finland, answered semi-structured questionnaires on their views on child feeding and health as well as their child’s diet quality. Participants were divided into two groups based on their self-perceived report of following a healthy diet: health-conscious (HC, *n* = 396) and non-health-conscious (non-HC, *n* = 342) parents. HC parents considered health, eating behavior, and nutrient-related factors more often when feeding their child than non-HC parents (<0.001 < *p* < 0.03). Moreover, they more commonly considered diet to have an important impact on their child’s long-term health than the non-HC parents (<0.001 < *p* < 0.05). Children of HC parents were more likely to have a good diet quality (*p* = 0.01) and lower BMI-SDS values (*p* = 0.015) than those of non-HC parents. Parental health consciousness was linked with better diet quality and healthier weight in their children. This information may be useful in the regular clinical monitoring of children’s health.

## 1. Introduction

Diet and physical activity habits develop during childhood and typically persist through to adulthood [1,2]. Therefore, the early establishment of health-promoting lifestyle habits is extremely important for the promotion of life-long health and for lowering the risk of chronic lifestyle-related diseases. Diet plays a role in the development of obesity, which is one of the most serious public health challenges of the 21st century globally [3]. It is estimated that as many as every third preschool-aged child in the US [4], and in Europe, almost one out of every four, have overweight or obesity [5]. The situation is similar in Finland, where 27% of boys and 16% of girls 2- to 6-years of age (preschool age in Finland) have overweight or obesity [6]. Furthermore, it should be emphasized that obesity tends to persist later in life [7].

As parents select and provide the foods for the family, contribute to the child’s feeding, and may also be present at family mealtimes, they have a major influence on the diets of preschool-aged children [8,9,10]. Thus, it is important to clarify the extent to which parental views on feeding are related to the dietary habits and susceptibility to developing obesity in their children. It has been suggested that the parents influence the development of their child’s eating behavior through the concept of positive parental role modeling: a parent’s own food preferences and eating behavior are adopted by their children and thus modify children’s dietary behavior and attitudes towards food [11,12,13,14]. Healthy-eating attitudes in the parents are known to affect the adequacy of children’s micronutrient intake in a positive manner, possibly even more than the parental knowledge of what represents a healthy childhood diet [15]. Moreover, previous findings indicate that parental knowledge of or attitudes towards a healthy diet as well as parents’ own dietary habits might be related to the child’s diet quality [16,17,18] and weight [19,20,21], but other investigators have also detected no association between some of these factors [15,16,22,23]. There is also evidence that parents might not understand how the childhood diet is linked with their child’s life-long health [24]. Furthermore, several information sources, such as health care professionals, family members, media, and advertising, might affect parental feeding practices [25,26,27,28,29], although this topic has mostly been studied in parents of small infants. Currently, there is a paucity of scientific evidence applying a comprehensive approach regarding parental views on child feeding practices and health in parents of preschool-aged children.

In this study, we investigated whether parents’ self-perceived appraisal that they had a healthy dietary intake, which is taken to reflect health consciousness (HC) as compared to non-HC parents, is reflected in the parental views on child feeding and health. We also assessed the parents’ views on the challenges and their ability to provide their children with a healthy diet. As quantitative measures, we investigated whether the diet quality, evaluated by a validated diet quality index [30], and weight in children differed between HC and non-HC parents.

## 2. Materials and Methods

### 2.1. Study Design and Participants

Data were collected in a cross-sectional study conducted in child health clinics across Finland from February to June 2016. Information on parental views on child feeding was collected by a semi-structured questionnaire modified from a format used by Gage and colleagues [24]. As the original questionnaire had been developed for assessing first-time mothers’ views on infant feeding, the questions were slightly modified, when necessary, so that they were appropriate for the feeding of older children. For example, age-specific questions regarding breastfeeding were not included. The forms were filled in by the guardian accompanying the child to the health clinic. In addition, information on background variables was collected through a questionnaire and the child’s diet quality was estimated by a validated index [30]. Nurses in the child health clinics measured the heights and weights of the children with the standard procedures used in Finnish child health clinics. The results were compared between HC and non-HC parents. The groups were determined with the following question: “to what extent do you follow a healthy diet” with response options ‘not at all’, ‘slightly’ or ‘moderately’ (non-HC parents), and ‘very much’ or ‘extremely lot’ (HC parents). The concept was decided to be extended from healthy eating to the parental health consciousness as other health-related parental characteristics supported this interpretation: HC parents were more likely to have a healthier weight status, a higher physical activity level, and a non-smoking status compared to non-HC parents (Table 1).

The recruitment process has been depicted in detail elsewhere [31]. In short, parents of children attending child health clinics for their annual health visits were asked to take part in the study. Eligible participants were parents with their 2- to 6-year-old children attending a child health clinic. A sufficient understanding of the Finnish language was required to ensure that they could fill in the questionnaire. Families with children who themselves were suffering from a severe disease, such as cancer, sequela of surgery, or were consuming a special diet for management of a disease, e.g., coeliac disease or allergy to cow’s milk, grains, or multiple foods, were excluded from the study. Furthermore, if the parent did not answer the question of whether the parent was following a healthy diet, the family was excluded from the present study. The study flow is depicted in Figure S1.

### 2.2. Questionnaires for Parents

A semi-structured questionnaire, using two separate 5-point Likert scales (not at all/slightly/moderately/very much/extremely or never/rarely/sometimes/usually/always) depending on the question, was designed to collect data on parental views on child feeding. Parents were asked about their views on the extent to which diverse information sources influence their decisions on child feeding and the extent to which they consider different factors when feeding their child. The factors were related to (1) the child’s health (e.g., the healthiness of food, effect on the child’s current health, effect on the child´s health in years to come, and possible symptoms caused by food), (2) eating behavior (e.g., ensuring that there are regular mealtimes, whether the family is eating together, the family’s and child’s food preferences), (3) nutrient composition of food (e.g., the content of sugar, fat, and fiber), and (4) food composition, appearance and preparation (e.g., food packaging, appearance, flavor and aroma, and whether food is homemade). Parents were also asked about their views on the extent to which the childhood diet can influence long-term health and specific diseases and conditions (e.g., risk of high blood pressure, type 2 diabetes, cancer, and cardiovascular diseases). Furthermore, the questionnaire inquired about parents’ views on the extent to which different factors (e.g., diet in adulthood, whether the child had been breastfed, and physical activity during childhood and adolescence) will affect the health of the child as she/he grows into adulthood. Parents’ views were also assessed on the extent to which they agree with several statements related to challenges regarding their ability to feed their child so that her/his lifelong risk of developing diseases would be reduced (e.g., providing a healthy diet is expensive, the child will not like a healthy diet, and other people sometimes care for my child). Lastly, the questionnaire inquired about parents´ views on their ability, probability, and willingness to feed their child always so that the child´s lifelong risk to develop a disease would be reduced. Examples of the questions and response options are shown in Table S1.

In addition, parents’ weights and heights, level of physical activity, and sociodemographic factors were collected by a questionnaire.

### 2.3. Child Diet Quality

The quality of the children’s diet in reference to the Finnish dietary recommendations was evaluated by the Children’s Index of Diet Quality (CIDQ), which has been previously validated in a population of Finnish 2- to 6-year-old children [30]. The questionnaire contains 15 structured questions on the quality and frequency of consumption of typical foods of the age group: porridge or gruel, fruits and berries, vegetables, spread, vegetable oil, cheese, milk, and sugary yogurts and juices. The CIDQ score ranged from 0 to 21 and was subsequently categorized into three groups: poor (<10 points), moderate (10.0–13.5 points), and good (≥14 points) diet quality as defined in the original validation study [30].

### 2.4. Weight and Height Measures

The children’s weights and heights were measured and recorded during the child health clinic visit by the nurses to an accuracy of 0.1 cm and 0.1 kg. These values were inverted to the BMI standard deviation score (BMI SDS) and categorized according to the Finnish growth reference curves [32]: underweight (BMI SDS ≤ −1.6482 for girls and BMI SDS ≤ −1.8344 for boys), normal weight (BMI SDS −1.6481–1.1628 for girls and BMI SDS −1.8343–0.7783 for boys), overweight (BMI SDS 1.1629–2.1064 for girls and BMI SDS 0.7784–1.7015 for boys), and obesity (BMI SDS ≥ 2.1065 for girls and BMI SDS ≥ 1.7016 for boys).

Parental self-reported weights and heights were used to calculate their body mass index (BMI) as kilograms per meter squared (kg/m^2^) and categorized into four weight classes according to the World Health Organization [33]: underweight (BMI < 18.5 kg/m^2^), normal weight (BMI 18.5–24.9 kg/m^2^), overweight (BMI 25.0–29.9 kg/m^2^), and obesity (BMI ≥ 30.0 kg/m^2^).

### 2.5. Statistical Analysis

A total of 770 parents answered the study questionnaires. Data from four participating families were excluded by the researchers since the inclusion criteria were not met and a further 28 answers were excluded due to the fact that they had not answered the question of whether the parent was following a healthy diet. Thus, the final number of responses was 738. The anthropometric data of eleven children were further excluded since their height standard deviation scores were ±2.7 SD, which was considered to be potentially due to recording errors or abnormal growth caused by some unknown disease.

The information in the questionnaires was transferred to Excel (Part of Microsoft Office Professional Edition, 2016) and analyzed with IBM SPSS Statistics (version 25.0 for Windows, IBM Corp, Armonk, NY, USA, 2017). Missing data were not imputed. The normal distribution of variables was visually examined from histograms. The Likert scale answers were pooled together, e.g., ‘not at all’ with ‘slightly’, and similarly ‘very much’ with ‘extremely’ to improve the interpretation of the results.

The differences in the views on child feeding and health between HC parents and non-HC parents were examined with ordinal logistic regression. Analyses were adjusted for parents’ gender, education, and whether the parent had a degree in the field of health or nutrition. Results are shown using adjusted odds ratios (OR) with 95% confidence intervals (CI). Chi-square tests were used to assess the differences in categorized background factors between HC parents and non-HC parents. Furthermore, the differences in children´s diet quality scores and BMI SDS values between the HC parent and non-HC parent groups were assessed with the Independent Samples T-Test. All analyses were 2-tailed and a *p*-value < 0.05 was considered significant.

## 3. Results

### 3.1. Descriptive Characteristics

Most of the responders (91.5%) were mothers; 8.5% were fathers. In most families, mothers (52.7%, 385/730) or mothers and fathers together (44.9%, 328/730) were in charge of the child´s diet. The father or someone else (partners, grandparent or grandparent together with a parent, family based daycare together with a parent) had the main responsibility for the child’s diet only in 1.5% (11/730) and 0.8% (6/730) of the families, respectively.

Of the responders, 53.7% (396/738) were considered HC parents and 46.3% (342/738) were non-HC parents. The mean BMI of the HC parents was lower than that of non-HC parents (24.3 ± 4.1 kg/m^2^ and 26.0 ± 4.9 kg/m^2^, respectively, *p* < 0.001). As shown in Table 1, HC parents also had a higher level of physical activity and were more commonly non-smokers than non-HC parents. Furthermore, HC parents had a higher level of education and more commonly a degree in a field of health or nutrition than non-HC parents. Most of the parents gave their child the recommended vitamin D supplement regularly 5 to 7 days per week, although this was more likely in the group of HC parents than in their non-HC counterparts. The vitamin D dose was mostly 10 µg, as recommended, in all families. The HC parents were also more likely than the non-HC parents to give their children other dietary supplements, such as multivitamins, minerals, and oil supplements (Table 1).

### 3.2. Child Diet Quality and Weight in Relation to Parental Health Consciousness

Children of the HC parents had better diet quality scores as evaluated by the CIDQ than those of non-HC parents (11.4 ± 2.6 and 10.6 ± 2.6, respectively). Children of HC parents were also more likely to have a good diet quality; the diet quality was good in 17% of the children of HC parents but only in 10% of the children of non-HC parents (Table 1).

The mean BMI SDS of children was within normal reference ranges in both HC and non-HC parent groups, but the BMI SDS values were significantly lower in children of the HC parents as compared to those of the non-HC parents (Table 1). However, no differences were detected between the groups in the children’s categorized BMI SDS with cut-offs from the Finnish growth charts. When using cut-offs by the International Task Force (IOTF) [34], the BMI SDS categories differed significantly between the HC and non-HC parents (Table 1).

### 3.3. Parents’ Views on Factors Considered When Feeding Their Child

**Figure 1:**
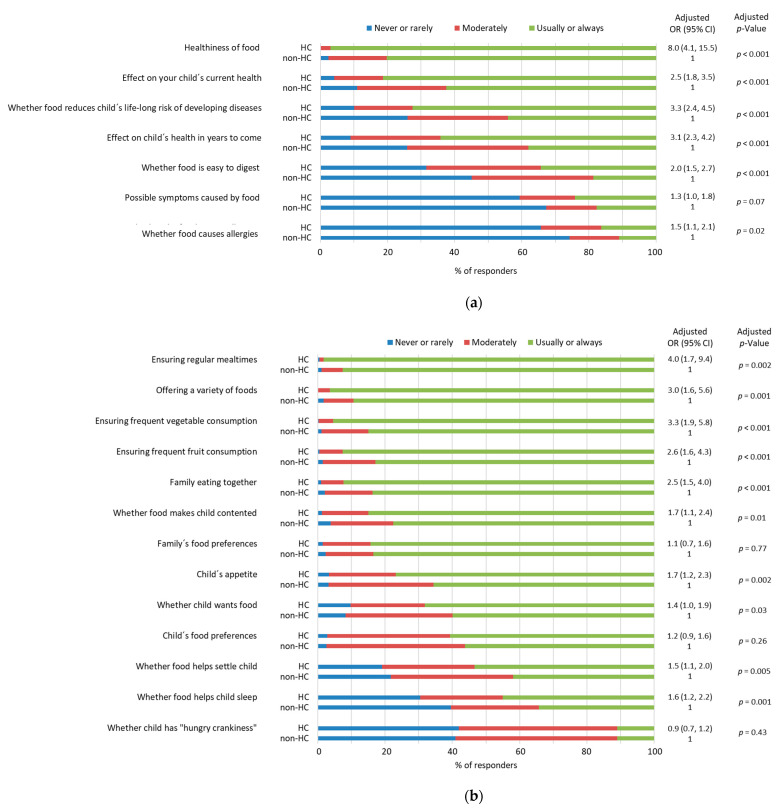

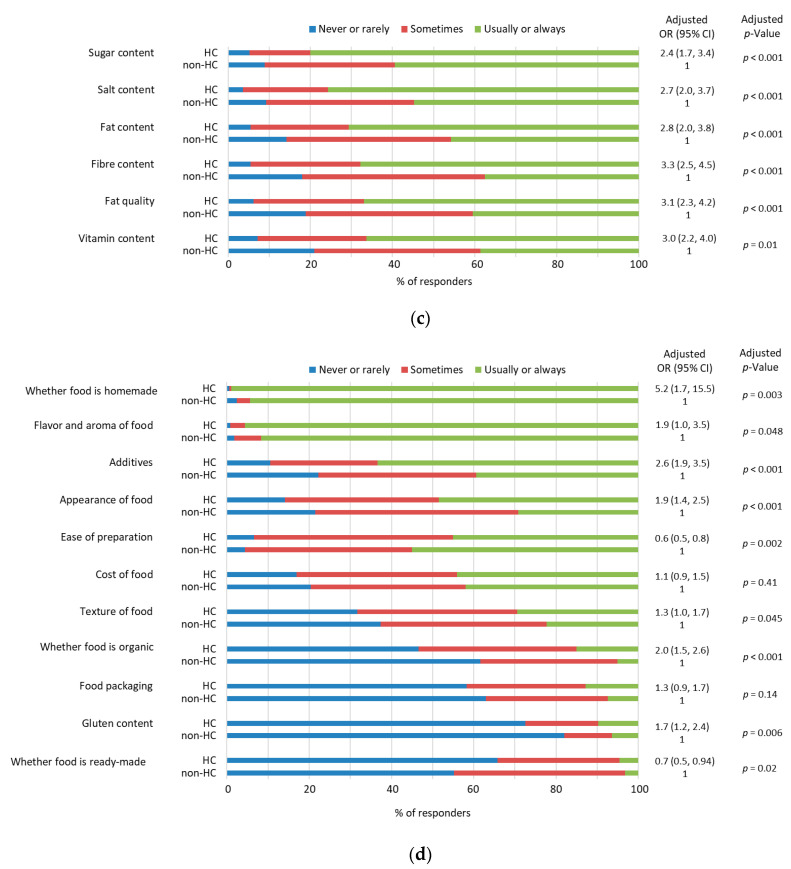
Associations between the parents’ views and parental health consciousness (HC = health-conscious and non-HC = non-health-conscious parents) examined by ordinal logistic regression analyses after adjustment for the parents’ gender, education, and whether the parent had a de-gree in a field of health or nutrition. OR = odds ratio, CI = confidence interval. (**a**) Views on the extent to which they consider factors related to health when feeding their child. Missing data *n* = 19–27 depending on the question. (**b**) Views on the extent to which they consider factors related to eating behavior when feeding their child. Missing data *n* = 16–27 depending on the question. (**c**) Views on the extent to which they consider factors related to the nutrient composition of food when feeding their child. Missing data *n* = 16–18 depending on the question. (**d**) Views on the extent to which they consider factors related to composition, appearance, and preparation of the food they give to their child. Missing data *n* = 17–19 depending on the question.

In general, when selecting meals for their child, HC parents were more likely to consider factors related to the child’s health, child´s eating behavior, and nutrient composition of food as well as the food’s composition, appearance, and preparation (Figure 1a–d). The ease of food preparation was more likely to be considered by the non-HC parents than the HC parents (Figure 1d). In more detail, HC parents more likely considered the effects of diet on the child´s current and long-term health, i.e., whether the food was associated with a reduction in the lifelong risk of developing diseases when the child was older, and the healthiness of the food as compared to non-HC parents (Figure 1a). While ensuring regular mealtimes and frequent consumption of vegetables and fruits by the child as well as eating together with the whole family emerged in both of the groups, it was more likely to be considered in HC parents (Figure 1b). Of the factors regarding the nutrient composition of food (Figure 1c), in both groups of parents, the sugar content of the foods they gave their child was most commonly considered. Of the factors related to food composition, such as its appearance and preparation (Figure 1d), the most often considered factor by all parents was whether the food was homemade as well as its flavor and aroma. Whether the food was organic or ready-made was considered rather rarely in both groups.

### 3.4. Parents’ Views on the Impact of Diet on Long-Term Health of Their Child

**Figure 2:**
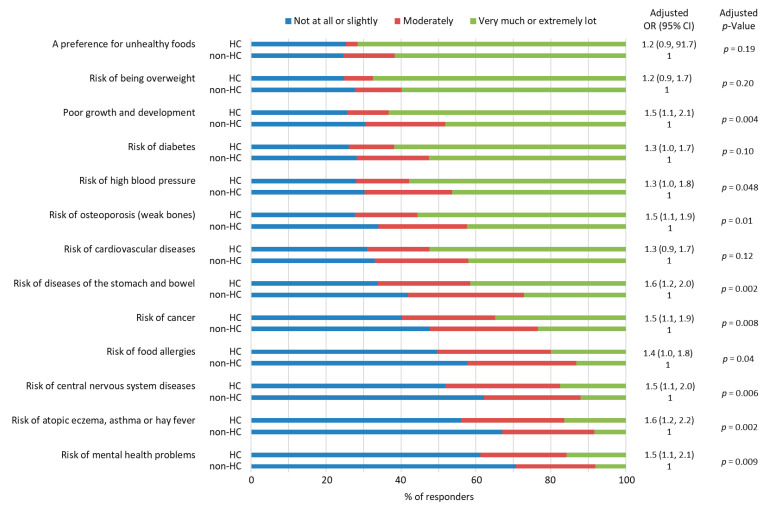
Health-conscious (HC) and non-health-conscious (non-HC) parents´ views on the extent to which childhood diet influences long-term health and specific diseases and conditions. Associations between parents’ views and parental health consciousness were examined by ordinal logistic regression analyses after adjustment for the parents’ gender, education, and whether the parent had a degree in a field of health or nutrition. Missing data *n* = 20–49 depending on the question. OR = odds ratio, CI = confidence interval.

HC parents were more likely to consider the child´s diet to be an important contributor to later health compared to non-HC parents (Figure 2). Parents in both groups stated that diet in childhood did influence the risk of developing a preference for unhealthy foods, the risk of being overweight, poor growth and development, and the risk of diabetes, whereas the most insignificant effects were perceived to be the risk of mental health problems, atopic eczema, asthma or hay fever, central nervous system diseases, and food allergies.

**Figure 3:**
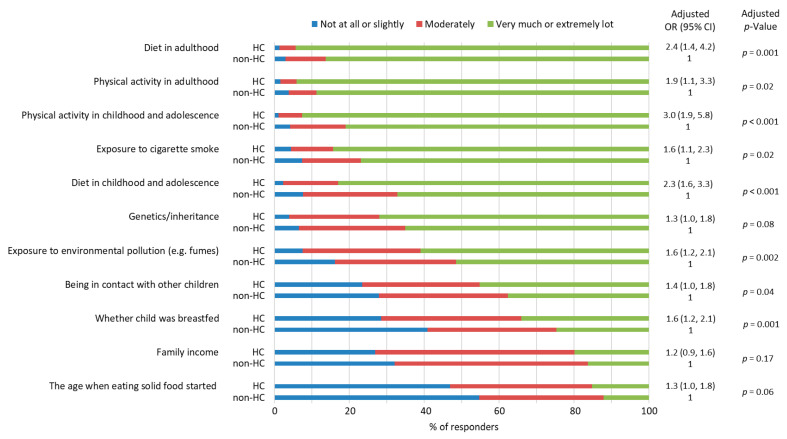
Health-conscious (HC) and non-health-conscious (non-HC) parents´ views on the extent to which different factors affect the health of the child in adulthood. Associations between the parents’ views and parental health consciousness were examined by ordinal logistic regression analyses after adjustment for the parents’ gender, education, and whether the parent had a degree in a field of health or nutrition. Missing data *n* = 31–43 depending on the question. OR = odds ratio, CI = confidence interval.

HC parents and non-HC parents both considered diet and physical activity in adulthood and physical activity in childhood and adolescence as the most important factors affecting the adulthood health of their child (Figure 3), although the HC parents were more likely to consider these factors to be significant. Furthermore, both parent groups evaluated the adulthood diet to be more effective in determining life-long health than the diet consumed in childhood and adolescence (HC parents were more likely than non-HC parents).

The early nutrition decisions, such as whether the child was breastfed and the age when eating solid food was started, in addition to family income were considered rather unimportant with respect to children´s adulthood health when compared to other factors (Figure 3).

### 3.5. The Information Sources Influencing Child Feeding Decisions

There were some significant differences between HC parents and non-HC parents regarding the information sources that influenced their child-feeding decisions (Figure S2a–c). HC parents were more likely to consider friends (adjusted OR 1.8, 95% CI 1.0−3.1, adjusted *p* = 0.04) and relatives, such as grandparents, (adjusted OR 1.8, 95% CI 1.0−3.1, adjusted *p* = 0.049) as information sources affecting their decisions on child feeding (Figure S2b). With respect to the information sources, intuition was the most significant; 46% of all parents reported intuition to influence very much or extremely on their decisions on how to feed their child (Figure S2c). In addition, the input from their partner affected the decisions about what type of food should be given to the child very much or extremely in 29% of all parents (Figure S2b). Additional factors other than intuition and partner were considered as less important: (1) the health and nutrition professional sources (child health clinic, midwife, nurse, doctor, or other health care personnel, e.g., dietician) were considered very much or extremely important by only 7–11% of parents, (2) relatives and acquaintances (e.g., grandparents, friends) by 2–12% of parents, and (3) other sources (e.g., written documents, social media, advertisements) by 0–7% of parents.

The overall effect of obtaining some kind of advice regarding child feeding from outside the immediate family was considered more often (26% considering them usually or always) than social media and advertisements (4% and 1%, respectively, considering them usually or always, Figure S3); the results did not differ between the HC and non-HC parents.

### 3.6. Parents’ Views on Challenges to Maintain A Healthy Diet in Their Children

**Figure 4:**
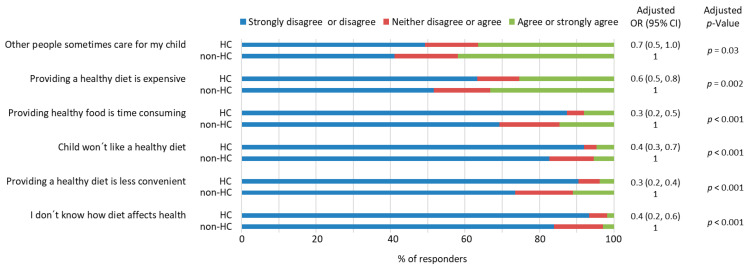
Health-conscious (HC) and non-health-conscious (non-HC) parents´ views on the challenges regarding their ability to feed their child so that her/his lifelong risk of developing disease would be reduced. Associations between the parents’ views and parental health consciousness were examined by ordinal logistic regression analyses after adjustment for the parents’ gender, education, and whether the parent had a degree in a field of health or nutrition. Missing data *n* = 35–38 depending on the question. OR = odds ratio, CI = confidence interval.

For both HC parents and non-HC parents, the most common challenges related to their ability to feed their child so that it would reduce her/his lifelong risk of developing diseases were that other people sometimes cared for their child and that providing healthy food was expensive (Figure 4). Most of the potential challenges to feeding their child with a healthy diet were considered as not relevant by both groups of parents (Figure 4).

Moreover, HC parents were more likely to consider the probability that their child was eating a diet that would reduce her/his lifelong risk of developing diseases than non-HC parents (adjusted OR 2.8, 95% CI 1.9−3.9, adjusted *p* < 0.001, Figure S4). This also applied to the likelihood of HC parents answering that they were willing that their child would have a diet that would reduce her/his lifelong risk of developing diseases (adjusted OR 2.1, 95% CI 1.2−4.0, adjusted *p* = 0.02) and that they are able to regulate their child’s feeding so that it would reduce their child´s lifelong risk of developing diseases (adjusted OR 2.6, 95% CI 1.9−3.6, adjusted *p* < 0.001, Figure S4).

## 4. Discussion

We found that HC parents considered both health and nutrition factors when feeding their child more often than their non-HC counterparts. Furthermore, HC parents were more aware of the long-term health effects of the childhood diet. Additionally, the diet quality was better and BMI SDS values were lower in children of HC parents as compared to those of non-HC parents.

In the present study, children of HC parents had better diet quality scores and were more likely to consume a good quality diet than those of non-HC parents, although the differences between the groups were small. Moreover, in a large Canadian study (*n* = 8388), it was reported that parents caring about a healthy diet was associated with a better quality diet being given to their child [16]. Similarly, Flemish’s (*n* = 862) and Omani’s (*n* = 154) studies on the parents of preschoolers also found that better nutritional knowledge and attitude scores in mothers were associated with better dietary scores in the children [17,18]. Moreover, in a Spanish study (*n* = 287), the children of parents with healthier eating attitudes had a better diet quality and a more favorable dietary intake than children of other parents; however, parental healthy-eating knowledge did not associate with the child’s diet [15]. Some investigators have also reported that parental health knowledge and beliefs displayed a positive association with single components in the diet consumed by their children, such as the consumption of vegetables [35] and milk [26,36].

We also observed that if the parent was following a healthy diet, then this was related to lower BMI SDS values in the child. Thus, our findings suggest that there could be a link between parental healthy eating behavior and the child’s weight, although, here too, the difference between the groups was rather small. This view is supported by findings from previous publications: a Greek study with 10–12-year-old children found that parents’ lower adherence to the Mediterranean diet was associated with their children being overweight [19], and a US study reported that the father’s higher energy intake was related to greater increases in their daughter’s BMI value between 5 and 7 years of age [21]. Furthermore, a Chinese study found a strong association between parental consumption of snacks and young children’s overweight [20]. However, some investigators have not detected an association between the child’s weight or BMI value and parental caring about a healthy diet [16], their attitudes to child feeding [22], or parental health literacy [23].

Interestingly, in our study, both parent groups believed that the diet consumed in adulthood would be more significant with respect to life-long health than the childhood diet. A similar result was found in the first-time mothers of infants in a similar questionnaire survey conducted in five European countries [24], whereas a systematic review of qualitative studies reported that parents believed that health-promoting habits should begin early in childhood to promote a long-term healthy lifestyle in their children as they grew older [37]. Most of the parents in our study intended to serve their children a healthy diet, and they believed that they possessed enough knowledge about what represented a healthy childhood diet. However, as we have previously reported from this data, an alarmingly low proportion of children had a good diet quality as assessed by a validated diet quality index [31]. Thus, it seems that parental health knowledge is not fully reflected in the practice of feeding their child, even though the parents’ own positive attitude regarding a healthy lifestyle is nevertheless an asset. It is noteworthy that parents also tend to overestimate the quality of their child´s diet as well as underestimate the child´s overweight status, i.e., their evaluation of their child’s health is not totally reliable [37,38]. Overall, a better understanding of the parents´ knowledge of what represents a healthy diet and how this is reflected in practice around the dinner table may be crucial to the evaluation of a child’s dietary habits. Furthermore, since the parents’ role in feeding their children decreases as the child grows older, it has been suggested that nutrition education in child health clinics should be targeted more strongly toward parents of preschool-aged children as opposed to older children [39]. Previous studies also suggest that primary prevention by promoting a healthy diet should be the main goal when targeting the correction of poor diet and the prevention of obesity in children, especially since modifying lifestyle habits may be difficult once obesity has become established [40]; targeting the preventive activities already towards the early childhood might be helpful.

In Finland, parents are recommended to visit the child health clinics regularly with their children and receive one-to-one guidance regarding health, diet, and physical activity [41]. We found that the parents considered the information given by healthcare professionals to be only a minor factor affecting how they feed their child, even though personal counseling has been reported to be highly appreciated by the parents of infants in Finland [42]. In previous studies, mainly conducted on parents of small infants, healthcare professionals have been important sources of information regarding child-feeding practices [26,27]. In fact, some studies have found that parents of younger children were more prone to rely on the information given by healthcare professionals when compared to those of older children [25,29]. Moreover, in an Australian study, pregnant women expecting their first child recognized healthcare professionals as a beneficial source of information regarding child feeding [28]. Unexpectedly, in our study, the parent’s own intuition was the strongest factor, which affected her/his feeding decisions. Factors influencing parental concepts regarding diet are known to be diverse, including social, economic, biological, and psychological factors [43]. The term “intuition” can be understood as a life-long comprehensive knowledge, and this may have been affected by the information received from health care professionals as well as from cultural influences and opinions of extended family members. Thus, it is possible that while intuitive knowledge may be similar to the information provided by healthcare professionals, in other families, it may be totally the opposite. In line with our findings, it has been previously suggested that the parent´s beliefs regarding the children´s diet are often gathered from non-professional sources, for example from family members and relatives [26,37]. However, also parental intuition has been previously claimed to be an important information source, although not the most important one [26,27]. Interestingly, in our study, social media was not considered an important source of information, regardless of the ubiquity of the smartphone and social media usage. Moreover, other external sources such as advertisements were not considered important, contrary to findings of a systematic review in which media and food advertising were considered important barriers to feeding a healthy diet to children as some parents felt it is difficult to know which foods were healthy [37].

The findings of the current study support the earlier conclusions about the effects of parental role modeling in child feeding. It has been suggested that improving parental attitudes so that they adopt healthy eating might be more effective in improving the child’s dietary habits through positive parental role modeling rather than only educating parents about what they should be serving their children at mealtimes [15]. According to our results, both HC and non-HC parents were willing to serve their children healthy food, but the proportion of children with a good diet quality was significantly lower among non-HC parents. One explanation for this finding could be that since non-HC parents do not seem to invest much effort in their own diet, they are also not acting as positive role models for their children regarding their eating habits and do not provide them with healthy food, which is subsequently reflected in their children’s diets. In Finland, the national recommendation for the children´s diet emphasizes that the whole family should eat together on a daily basis [44], a behavior that may enforce positive parental role modeling regarding healthy eating habits [14]. The recommendation seemed to be reflected in the results of the present study, as the parents stated that eating together around the dining table was important. It has been suggested that interventions aimed at promoting a healthy diet in the parents may have a greater impact on the children´s diet quality, e.g., by modulating children´s thinking and attitudes, than interventions focusing exclusively on the children´s diet, as their impact may be more transient [2,11,13,14].

The large sample size is a notable strength of this study. We recruited parents and children from child health clinics all around the country and consider the sample to be representative of the Finnish population; firstly, since the proportions of children that were overweight or obese were similar to the official statistics of the same age group in Finland [6]. Secondly, in our respondents, there was also an adequately even distribution in terms of the children´s ages and the proportions of boys (44.9%) and girls (55.1%). As a potential limitation, the non-Finnish speaking parents were excluded since the questionnaire was only in the Finnish language. In Finland, the proportion of foreign-language speakers was 6.4% in 2016 [45]. In addition, the number of university-educated respondents was higher than the Finnish average; 30% of Finnish adults received a university education in 2016 [46], whereas the proportion in our study was 48%. However, this seems to be a universal trend in voluntary surveys. It is also considered a strength that we have taken a comprehensive approach to studying the parental views on child feeding, a topic evaluated with a variety of methods. As always in surveys, there is still a possibility that the respondent comprehends the questions differently than the researcher meant them to be understood. Further, the questionnaire was not validated for the purpose of this study. However, a similar questionnaire, with only slight modifications, was used earlier in a multinational survey [24] focusing on the views on infant feeding of first-time mothers. Furthermore, the grouping of the parents was based on the parents’ self-perceived healthy dietary intake, and we do not have exact information about the actual healthiness of their diet. Nevertheless, the inner validation supports the veracity of that self-perceived information as significant differences were seen between the HC and non-HC parent groups also in other health-related supportive characteristics, such as in weight status, physical activity, and smoking status. Thus, we considered this approach to be an acceptable way to expand the concept from simply eating a healthy diet to being health-conscious. Lastly, the data were collected in 2016 and it must be acknowledged that lifestyle habits may have changed since that date due to various reasons, such as the COVID-19 pandemic.

## 5. Conclusions

Our study adds to the previous knowledge indicating that parents´ views and practices regarding their child´s feeding and health are affected by parents’ health consciousness. The results also suggest that these factors may further affect the quality of the diet that they provide to their children and the child’s weight. This information may be useful in the regular clinical monitoring of children’s health. A more concise evaluation of both children´s and parents´ diet quality in child health clinics could be beneficial in identifying those children that are at risk of developing diet-related health conditions later in life. Using a validated diet quality index in children complemented with a question regarding parental adherence to a healthy diet may provide enough information about a family’s eating habits in order to identify those families most in need of dietary counseling. The results suggest that dietary counseling in child health clinics should be directed towards the whole family; positive parental role modeling should be emphasized, e.g., encouraging parents to pay attention to their own diet may help in improving the quality of the food consumed by their child. Furthermore, an open and more detailed dialogue about parental food-related attitudes could be helpful.

## Figures and Tables

**Table 1:** Parental and child characteristics of all participants (*n* = 738) and in groups of health-conscious (HC) and non-health-conscious (non-HC) parents.

		All		HC Parents		Non-HC Parents		
	Total *n* ^a^	*n* or Mean	% or SD	*n* or Mean	% or SD	*n* or Mean	% or SD	*p*-Value
**Parental characteristics**								
**Sex**	732							0.05 **^b^**
Mother		670	91.5	367	93.4	303	89.4	
Father		62	8.5	26	6.6	36	10.6	
**Age (years) ^b^**	737							0.99 **^b^**
<30		141	19.1	75	19.0	66	19.3	
30–34.9		250	33.9	134	33.9	116	33.9	
35–39.9		215	29.2	114	28.9	101	29.5	
≥40		131	17.8	72	18.2	59	17.3	
**Overweight status ^b^**	717							<0.001 **^b^**
Underweight		17	2.4	7	1.8	10	3.0	
Normal weight		391	54.5	239	62.4	152	45.5	
Overweight		200	27.9	97	25.3	103	30.8	
Obesity		109	15.2	40	10.4	69	20.7	
**Physical activity ^b^**	734							<0.001 **^b^**
Not at all or very little		500	68.1	203	51.4	297	87.6	
Moderately		201	27.4	162	41.0	39	11.5	
Very much or extremely		33	4.5	30	7.6	3	0.9	
**Education ^b^**	733							0.05 **^b^**
Comprehensive school/ upper secondary school/ vocation school		378	51.6	189	48.2	189	55.4	
University of applied sciences/ university education		355	48.4	203	51.8	152	44.6	
**Household income (€)**	700							0.16 **^b^**
≤40,000		232	33.1	115	30.3	117	36.6	
40,001–60,000		221	31.6	121	31.8	100	31.3	
>60,000		247	35.3	144	37.9	103	32.2	
**Smoking ^c^**	736	106	14.4	40	10.1	66	19.4	<0.001 **^b^**
**Degree in a field of health or nutrition ^c^**	717	241	33.6	143	37.1	98	29.4	0.03 **^b^**
**Child’s characteristics**								
**Sex**	738							0.72 ^b^
Boy		331	44.9	180	45.5	151	44.2	
Girl		407	55.1	216	54.5	191	55.8	
**Age (years)**	738							0.44 ^b^
2		162	22.0	79	19.9	83	24.3	
3		130	17.6	70	17.7	60	17.5	
4		154	20.9	80	20.2	74	21.6	
5		149	20.2	82	20.7	67	19.6	
6		143	19.4	85	21.5	58	17.0	
**BMI SDS values**	694	0.08	1.11	−0.02	1.12	0.19	1.09	0.015 ^d^
**BMI SDS categories** **according to the Finnish growth charts**	694							0.25 ^b^
Underweight		30	4.3	18	4.9	12	3.7	
Normal weight		529	76.2	289	77.9	240	74.3	
Overweight		106	15.3	53	14.3	53	16.4	
Obesity		29	4.2	11	3.0	18	5.6	
**BMI SDS categories** **according to the IOTF criteria ^e^**	694							0.002 ^d^
Underweight		113	16.3	69	18.6	44	13.6	
Normal weight		488	70.3	255	68.7	233	72.1	
Overweight		74	10.7	44	11.9	30	9.3	
Obesity		19	2.7	3	0.8	16	5.0	
**Diet quality (CIDQ) scores**	710	11.1	2.7	11.4	2.6	10.6	2.6	<0.001 ^d^
**Diet quality (CIDQ) categories**	710							0.01 ^b^
Poor		215	30.3	104	26.9	111	34.3	
Moderate		397	55.9	217	56.2	180	55.6	
Good		98	13.8	65	16.8	33	10.2	
**Use of vitamin D supplement**	725							0.03 ^b^
0–2 times in a week		44	6.1	20	5.2	24	7.1	
3–4 times in a week		70	9.7	28	7.2	42	12.5	
5–7 times in a week		611	84.3	340	87.6	271	80.4	
**Use of other supplements ^f^**	727	266	36.6	169	43.0	97	29.0	<0.001 ^b^
**Has a health condition ^g^**	738	70	9.5	39	9.8	31	9.1	0.53 ^b^

BMI SDS, Body Mass Index Standard Deviation Score. CIDQ, Children’s Index of Diet Quality. IOTF, International Obesity Task Force. SD, standard deviation. ^a^ A maximum of 44 missing answers depending on the question. ^b^ Differences between the groups of health-conscious parents (HC) and non-health-conscious parents (non-HC), Chi-Squared Test. ^c^ Information from the parent responding to the questionnaire (91.5% of responders were mothers and 8.5% fathers). ^d^ Differences between the health-conscious parent (HC) and non-health-conscious parent (non-HC) groups, Independent Samples T-test. ^e^ BMI SDS cut-offs according to the IOTF criteria: BMI SDS ≤ −0.976 for girls and BMI SDS ≤ −1.015 for boys (underweight), BMI SDS −0.975–1.243 for girls and BMI SDS −1.014–1.309 for boys (normal weight), BMI SDS 1.244–2.191 for girls and BMI SDS 1.310–2.287 for boys (overweight), and BMI SDS ≥ 2.192 for girls and BMI SDS ≥ 2.288 for boys (obesity). ^f^ Other supplements including children’s multivitamin and mineral supplements, cod liver oil, or probiotics. ^g^ Health conditions including asthma, atopic eczema, or allergy (pollen, animals, foods) (*n* = 54), disorders affecting central nervous system (*n* = 5), internal disorder (*n* = 14), or others (*n* = 6). Children with a severe disease or a special diet with connection to disease or food allergy, that would exert a major impact on the children’s diet, were excluded.

## Data Availability

The data are not publicly available as they contain information that could compromise the privacy of the research participants.

## Supplementary Materials

The following supporting information can be downloaded at: https://www.mdpi.com/article/10.3390/nu15041024/s1, Figure S1. Study flow chart. Figure S2a: Health-conscious (HC) and non-health-conscious (non-HC) parents’ views on the extent to which healthcare professionals as information sources affect decisions regarding their child’s feeding practices; Figure S2b: Health-conscious (HC) and non-health-conscious (non-HC) parents’ views on the extent to which relatives and acquaintances act as information sources which affect their decisions regarding the child’s feeding practices; Figure S2c: Health-conscious (HC) and non-health-conscious (non-HC) parents’ views on the extent to which other information sources affect their decisions regarding the child’s feeding practices; Figure S3: Health-conscious (HC) and non-health-conscious (non-HC) parents’ views on the extent to which they consider diverse information sources when feeding their child. Figure S4: Health-conscious (HC) and non-health-conscious (non-HC) parents’ views on the extent of their probability, ability, and willingness to feed their child so that the lifelong risk of developing a disease would be reduced. Table S1. Examples of the study questions and response options.
