# Supplementary material for: Self-Reported Parental Healthy Dietary Behavior Relates to Views on Child Feeding and Health and Diet Quality
Source: Nutrients. 2023 Feb 18;15(4):1024. doi: 10.3390/nu15041024 (PMC9959008; doi:10.3390/nu15041024)
Supplement: Supplementary file 1 [file nutrients-15-01024-s001.zip › nutrients-2155429-supplementary.pdf]

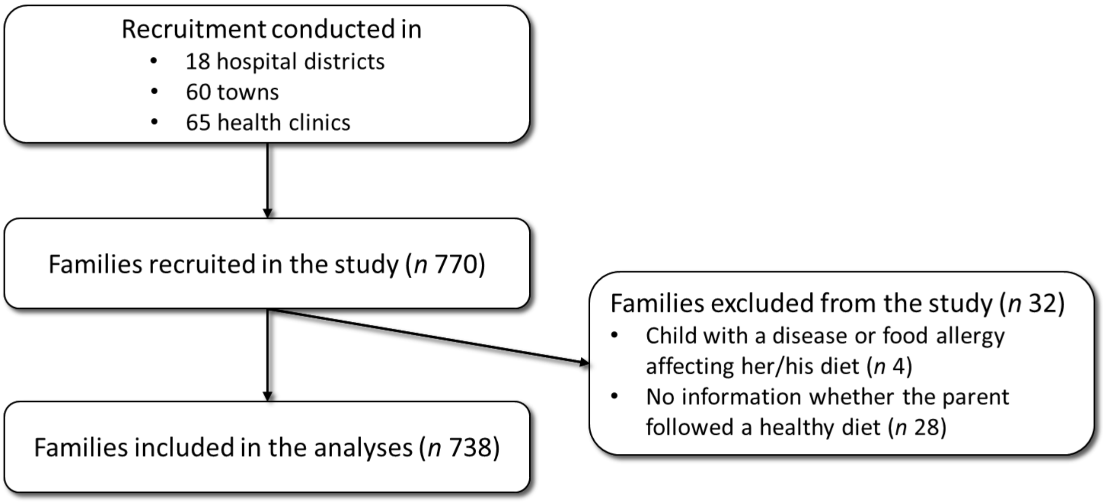

Figure S1: Study flow chart.

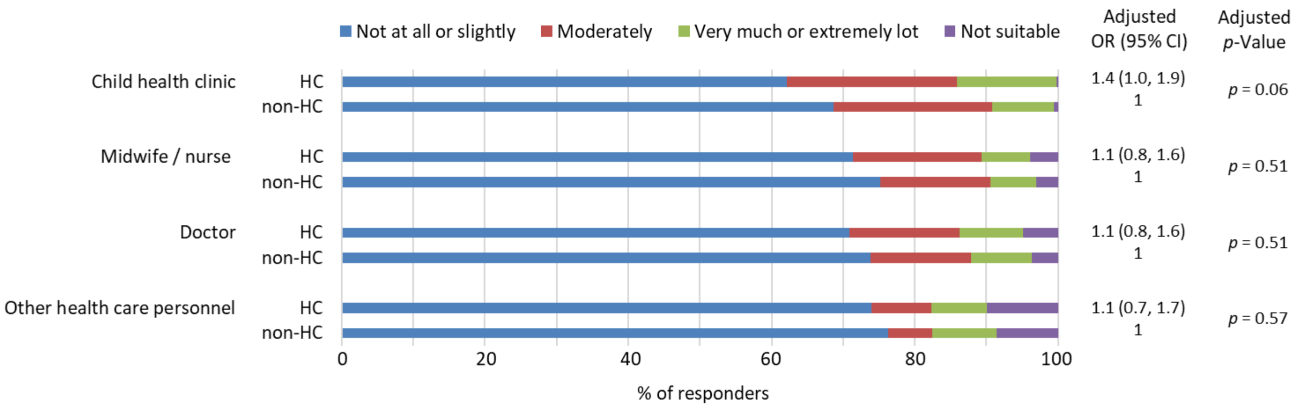

Figure S2a: Health-conscious (HC) and non-health-conscious (non-HC) parents' views on the extent to which health care professionals as information sources affect decisions regarding their child`s feeding practices. Associations between parents' views and parental health consciousness were examined by ordinal logistic regression analyses after adjustment for parents' gender, education and whether the parent had a degree in a field of health or nutrition. 'Not applicable' answers were excluded from the ordinal logistic regression analyses. OR=odds ratio, CI=confidence interval.

Mäkelä *et al*. 2023. Self-reported parental healthy dietary behavior relates to views on child feeding and health and diet quality. *Nutrients.*

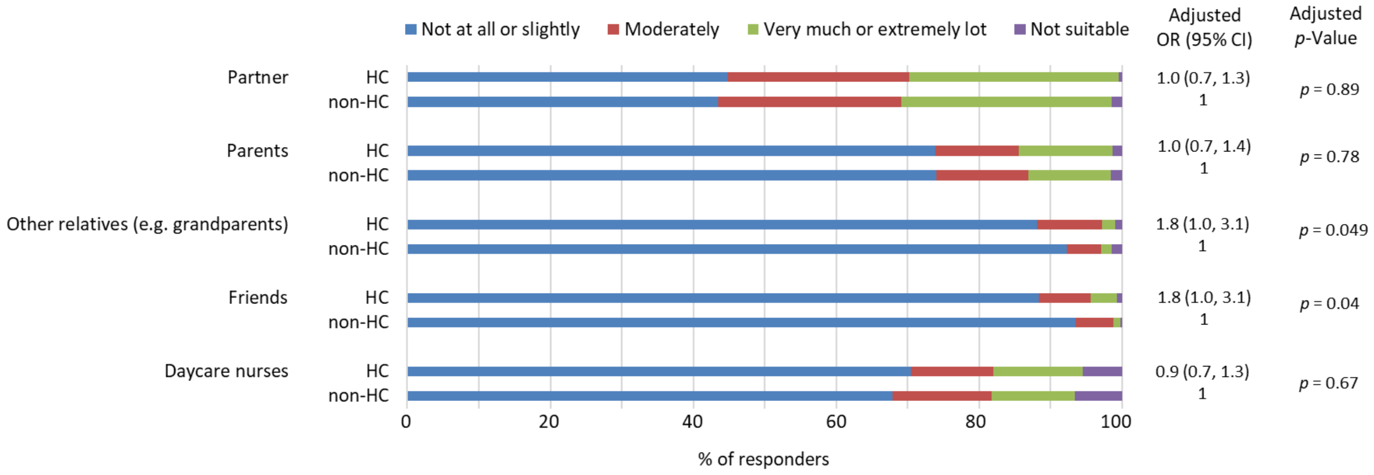

Figure S2b: Health-conscious (HC) and non-health-conscious (non-HC) parents' views on the extent to which relatives and acquaintances act as information sources which affect their decisions regarding the child`s feeding practices. Associations between parents' views and parental health consciousness were examined by ordinal logistic regression analyses after adjustment for parents' gender, education and whether the parent had a degree in a field of health or nutrition. 'Not applicable' answers were excluded from the ordinal logistic regression analyses. OR=odds ratio, CI=confidence interval.

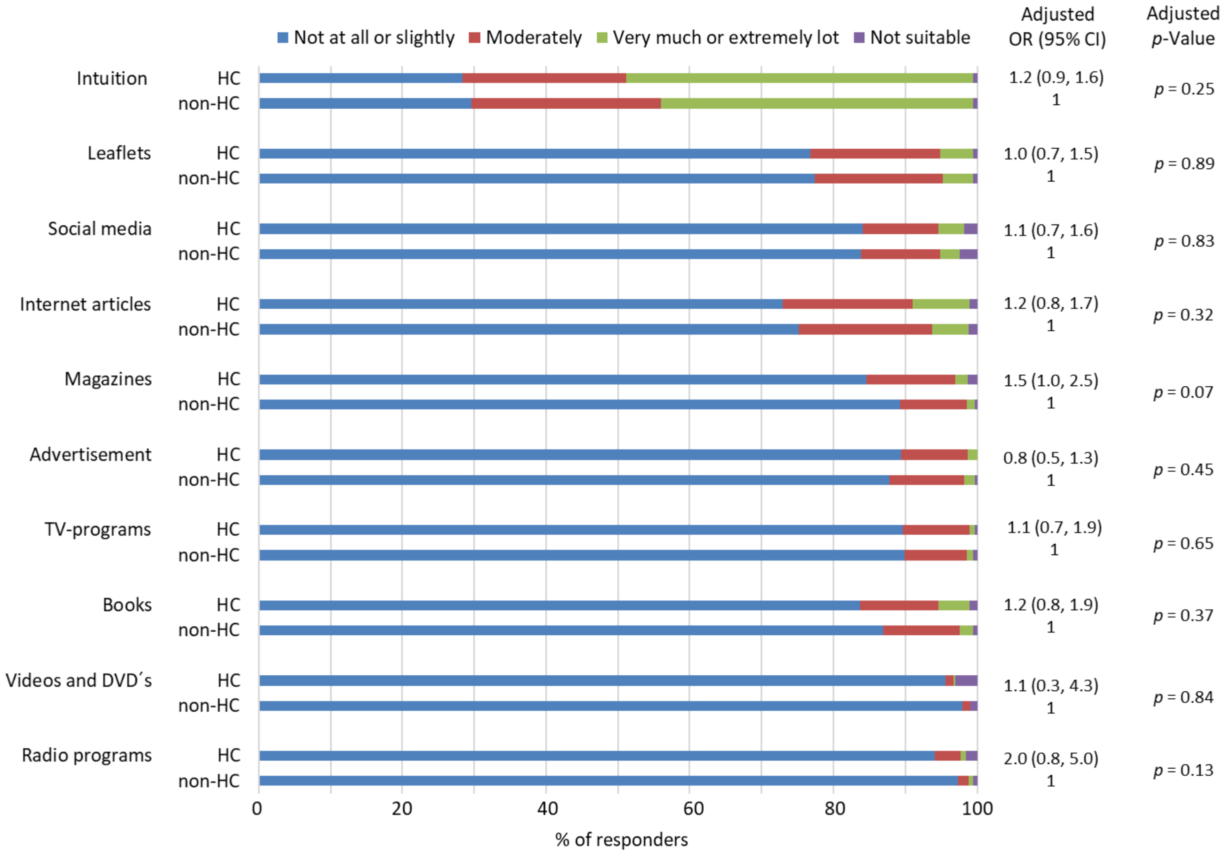

Figure S2c: Health-conscious (HC) and non-health-conscious (non-HC) parents' views on the extent to which other information sources affect their decisions regarding the child`s feeding practices. Associations between parents' views and parental health consciousness were examined by ordinal logistic regression analyses after adjustment for parents' gender, education and whether the parent had a degree in a field of health or nutrition. 'Not applicable' answers were excluded from the ordinal logistic regression analyses. OR=odds ratio, CI=confidence interval.

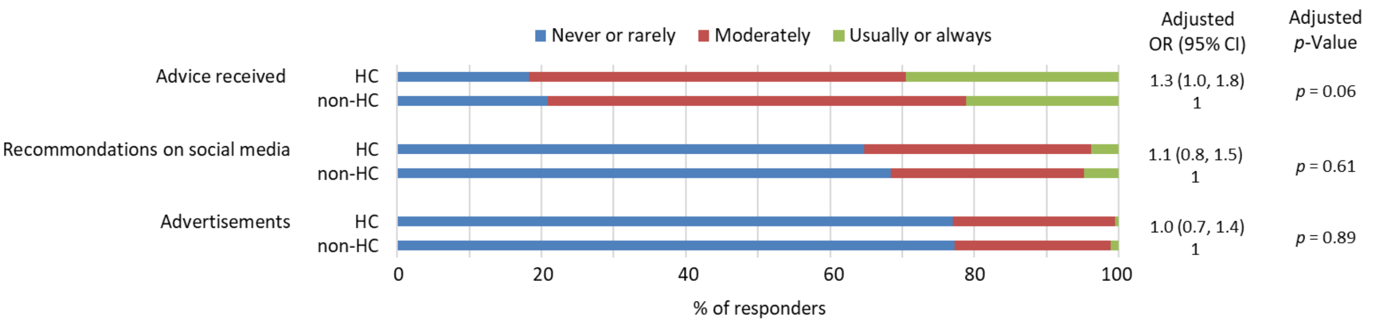

Figure S3: Health-conscious (HC) and non-health-conscious (non-HC) parents' views on the extent to which they consider diverse information sources when feeding their child. Associations between parents' views and parental health consciousness were examined by ordinal logistic regression analyses after adjustment for parents' gender, education and whether the parent had a degree in a field of health or nutrition. OR=odds ratio, CI=confidence interval.

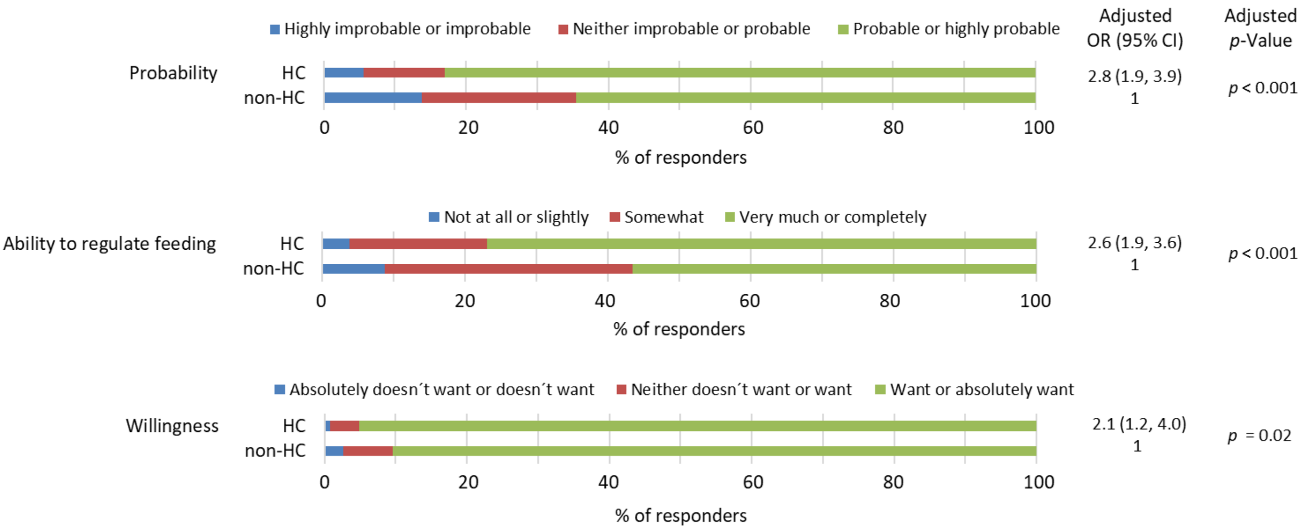

Figure S4: Health-conscious (HC) and non-health-conscious (non-HC) parents' views on the extent of their probability, ability, and willingness to feed their child so that the lifelong risk of developing a disease would be reduced. Associations between parents' views and parental health consciousness were examined by ordinal logistic regression analyses after adjustment for parents' gender, education and whether the parent had a degree in a field of health or nutrition. OR=odds ratio, CI=confidence interval.

Table S1. Examples of the study questions and response options.

**Question**	**Response options**					
**When feeding your child, how often do you consider the following?**	Never	Rarely	Sometimes	Usually	Always	
Whether food is homemade						
Whether food is easy to digest						
Cost of food						
**To what extent do you think the child's diet can influence her/his long-term health?**	Not at all	Slightly	Moderately	Very much	Extremely lot	
Poor growth and development						
Risk of being overweight						
Risk of food allergy						
**To what extent do you think the following factors will affect the health of the child in adulthood?**	Not at all	Slightly	Moderately	Very much	Extremely lot	
Genetics/inheritance						
Diet in childhood and adolescence						
Exposure to cigarette smoking						
**To what extent do you agree with the following statements?**<br>**My ability to feed my child, so that her/his lifelong risk of developing diseases would be reduced, is decreased as…**	Strongly disagree	Disagree	Neither disagree or agree	Agree	Strongly agree	
Other people sometimes care for my child						
Providing a healthy diet is expensive						
Providing healthy food is time consuming						
**To what extent the following factors influence on your decisions on child feeding?**	Not at all	Slightly	Moderately	Very much	Extremely lot	Not applicable
Intuition						
Partner						
Child health clinic personnel						
